# The Presence of Emphysema in Patients with Idiopathic Pulmonary Fibrosis and Lung Cancer: Impact on Tumor Features, Acute Exacerbation, and Survival

**DOI:** 10.3390/jcm14113862

**Published:** 2025-05-30

**Authors:** Xiaoyi Feng, Wenjing Zeng, Xiafei Lv, Binmiao Liang, Xuemei Ou

**Affiliations:** 1 Department of Respiratory and Critical Care Medicine, Peking Union Medical College Hospital, Chinese Academy of Medical Sciences & Peking Union Medical College, Beijing 100730, China; fengxiaoyi1997@163.com; 2 Department of Pulmonary and Critical Care Medicine, West China Hospital, Sichuan University, Chengdu 610041, China; 19914695418@163.com (W.Z.); liangbinmiao@163.com (B.L.); 3 Department of Radiology, West China Hospital, Sichuan University, Chengdu 610041, China; lvxiafei@163.com

**Keywords:** idiopathic pulmonary fibrosis, combined pulmonary fibrosis and emphysema, overall survival, acute exacerbation, lung cancer, epidermal growth factor receptor mutation, emphysema

## Abstract

**Background:** Idiopathic pulmonary fibrosis (IPF) and emphysema often coexist in patients with lung cancer (LC), forming a syndrome with combined pulmonary fibrosis and emphysema (CPFE). The three share the pathogenic mechanisms of smoking, chronic inflammation, and oxidative stress. The clinical management of CPFE patients is challenging, but its impact on tumor characteristics, acute exacerbation (AE), and prognosis is still controversial. The purpose of this study was to clarify the effect of CPFE on tumor biological behavior, AE risk, and survival outcome in patients with IPF-LC so as to optimize individualized treatment strategies. **Methods:** This was a retrospective and single-center study. Newly diagnosed LC patients with IPF, COPD, and normal lungs were recruited in the west China hospital. Patients with IPF were further categorized into CPFE-LC and isolated IPF-LC groups based on the presence of emphysema. Clinical and tumor features, lung function parameters, and prognosis were obtained and compared. **Results:** Patients with IPF and LC were more common in older men and heavy smokers. IPF-associated tumors had a higher proportion of carrying EGFR wild-type, occurring in the lower lobe of the lung and developing adenocarcinoma and squamous cell carcinoma. Among IPF-LC patients, 68.2% (103/151) met CPFE criteria. Pulmonary function tests demonstrated preserved VC% but significantly reduced FEV1/FVC in CPFE versus non-emphysema IPF (76.3% vs. 80.7%, *p* = 0.004), alongside elevated CPI and impaired DLCO. CPI ≥ 40 (HR = 2.087, 95%CI: 1.715–6.089, *p* = 0.012), combined with COPD (HR = 2.281, 95%CI: 1.139–4.569, *p* = 0.040), isolated IPF (HR = 5.703, 95%CI: 2.516–12.925, *p* < 0.001), and CPFE (HR = 6.275, 95%CI: 3.379–11.652, *p* < 0.001), were independent prognostic risk factors in LC patients. The incidence of treatment-induced AEs (49.5% vs. 29.2%, *p* = 0.038) and AE-related mortality (28.0% vs. 11.8%, *p* = 0.045) were significantly higher in the CPFE group than in the isolated IPF group. Logistic regression analysis showed that CPFE (OR: 3.494, 95%CI: 2.014–6.063, *p* = 0.001) was independently associated with the risk of AE-related mortality in patients with LC and IPF. **Conclusions:** Compared to LC patients with solely IPF, the presence of emphysema had no significant impact on overall survival, but CPFE increased the risk of treatment-triggered AE and was associated with AE-related mortality. In patients with LC, CPFE with AEs had a worse prognosis than IPF with AEs.

## 1. Introduction

Lung cancer (LC) remains the leading cause of cancer-related mortality worldwide. According to the latest epidemiological data (GLOBOCAN 2020), approximately 2.20 million new lung cancer cases were diagnosed globally in 2020, accounting for 11.4% of all newly diagnosed malignancies worldwide, with 1.79 million deaths attributed to the disease (constituting 18.0% of total cancer-related mortality) [1,2]. Chronic obstructive pulmonary disease (COPD) is the third leading global cause of death, afflicting approximately 10.3% of individuals aged ≥ 40 years. Emphysema, a key COPD phenotype, occurs in 20–30% of chronic smokers on HRCT [3]. Idiopathic pulmonary fibrosis (IPF)—a rare interstitial lung disease (2–29/100,000 prevalence)—has seen rising global incidence and carries a poor prognosis with 3–5 year median survival [4,5].

Emphysema often appears in patients with interstitial lung disease (ILD) with fibrosis, which is called combined pulmonary fibrosis and emphysema (CPFE) [6,7]. CPFE occurs in 26–54% of patients with idiopathic interstitial pneumonia, with a higher incidence in patients requiring hospitalization [8]. It has been reported that CPFE patients with usual interstitial pneumonia (UIP) have an approximately 2.69-fold increased risk of developing LC compared to patients with idiopathic pulmonary fibrosis (IPF) alone [9]. In clinical practice, it is critical to distinguish CPFE-LC, which has significantly lower treatment tolerance and higher risk of acute exacerbation (AE) and mortality, and IPF-LC, which requires priority for inhibiting the progression of fibrosis and rigorous evaluation of the safety of antitumor therapy [10,11]. A clear differentiation between the two can optimize individualized treatment and risk stratification, thereby improving prognosis and reducing the risk of complications.

So far, there has not been a consensus on the effect of emphysema on the prognosis of IPF patients combined with LC. A few studies have reported that the presence of emphysema significantly reduced the survival time of IPF patients with LC [12,13]. Several retrospective studies have contradicted this theory by reporting that the presence and extent of emphysema had no prognostic effect on survival in patients with IPF after adjusting for baseline disease severity. They believed that CPFE cannot be recognized as an independent prognostic factor for LC patients with IPF [14,15,16]. Nevertheless, other researchers have indicated that IPF patients with more extensive emphysema have better survival than IPF patients without emphysema [17].

The natural history of IPF has great heterogeneity, ranging from chronic stable condition to progressive respiratory failure or AE. The incidence of AE in IPF is 5–10% per year, and the presence of LC raises the risk of AE. Available studies suggest that the incidence of AE varies by race [18,19]. AE can lead to adverse events such as acute respiratory distress syndrome (ARDS), requiring hospitalization or even ICU treatment, which greatly increases the risk of death. Sato et al. reported long-term survival in patients with ILD and LC. Of 1763 patients, 378 died of cancer, 72 died of acute deterioration within 30 days after surgery, and 117 died of AE [20]. Several studies have shown that the risk of AE caused by oncologic treatments such as chemotherapy and surgery is significantly increased in patients with CPFE and LC [15,16,21]. To date, the impact of emphysema on the occurrence of CPFE-related AE (both natural process-related AE and treatment-induced AE) and AE-related mortality has not been well elucidated. More importantly, some studies have found that the prognosis of AEs of CPFE may be better than that of IPF alone [17,22], but to the best of our knowledge, little attention has been paid to whether the prognosis of AEs occurring in CPFE differs from that of IPF alone in LC patients.

Although the coexistence of lung cancer and ILD has been gradually recognized, the impact of the overlap of emphysema and pulmonary fibrosis on the tumor characteristics, risk of AE, and survival of patients with LC has not been clarified. In this study, we investigated for the first time the impact of CPFE on AE-related lethality and prognosis in LC patients and found significant differences in EGFR mutation rates in patients with IPF combined with adenocarcinoma, which provides a new direction for an in-depth understanding of the molecular mechanisms and clinical management of this disease.

## 2. Materials and Methods

### 2.1. Study Population

This is retrospective and observational research, approved by the Biomedical Ethics Review Committee of West China Hospital of Sichuan University (No. 2021-1374). One hundred fifty-one patients diagnosed with IPF and LC who were admitted to the Department of Respiratory Medicine at West China Hospital from October 2015 to September 2021 were enrolled in the study, called the IPF-LC group (*n* = 151), and they were further divided into the CPFE-LC group (*n* = 103) and the isolated IPF-LC group (*n* = 48) based on the presence of emphysema on chest high-resolution computed tomography (HRCT). Meanwhile, 99 patients with COPD and emphysema observed by chest CT, defined as the solely emphysema group (Em-LC group, *n* = 99), and 101 LC patients with normal lungs (Norm-LC group, *n* = 101) hospitalized during the same period were matched with adjustment of the pathological stage and included in this study.

### 2.2. Definitions of CPFE, IPF, and COPD with Emphysema

Patients were classified into the following categories based on chest HRCT and pulmonary lung functions. (1) CPFE: According to Cottin et al. [7], upper lobe emphysema of any subtype is defined as well-demarcated areas of low attenuation (CT value < 910 HU) delimitated by a very thin wall (<1 mm) or no wall, and emphysema area/total lung volume > 5%. The lower lobe showed lung fibrosis of any type. In this study, only pulmonary fibrosis in the pattern of usual interstitial pneumonia (UIP) was included. The UIP, including typical grid shadow, honeycombing, and traction bronchiectasis in subpleural and lower lung distribution, was diagnosed on the presence of a definite or probable UIP pattern based on the 2018 ATS/ERS/JRS/ALAT official IPF diagnosis and management guidelines [23]. (2) IPF: The presence of UIP and the absence of significant emphysema (emphysema area/total lung volume < 5%). (3) COPD with emphysema: COPD was defined as a predicted forced expiratory volume in 1 s (FEV1)/forced vital capacity (FVC) value ≤ 70% according to the criteria developed by the Global Initiative on Chronic Obstructive Pulmonary Disease (GOLD) report, and emphysema was observed by chest HRCT [24].

Exclusion criteria: (1) <18 years old; (2) patients with incomplete clinical data; (3) patients with patterns of fibrosis different from UIP; (4) patients with connective tissue disease (CTD) and any other ILDs such as sarcoidosis, pulmonary histiocytosis, eosinophilic pneumonia, hypersensitivity pneumonitis and occupational lung diseases, autoimmune disease, pure asthma, severe cardiovascular and cerebrovascular diseases, hematologic tumor, and patients without confirmed primary lung cancer; (5) patients who were lost to follow-up.

### 2.3. Diagnosis of Lung Cancer

All patients with lung cancer were diagnosed by pathological examination or surgery. Staging of lung cancer was performed in accordance with the 2015 National Comprehensive Cancer Network guidelines.

### 2.4. Definition of AE

AE was defined if they satisfied all the following criteria: (1) acute exacerbation of sudden respiratory failure within 30 days; (2) newly developed bilateral ground-glass opacity and/or consolidation superimposed on a background pattern consistent with UIP pattern on chest CT and/or chest X-ray; (3) decrease in arterial oxygen tension (PaO_2_) of more than 10 mmHg under similar conditions; and (4) absence of other known causes such as heart failure or fluid overload of deteriorating respiratory function. Treatment-induced AEs included acute deterioration triggered by anti-cancer drugs (carboplatin/etoposide, carboplatin/paclitaxel/bevacizumab, gefitinib) (within 4 weeks after treatment) and post-surgery (within 12 months after surgery), otherwise defined as AEs associated with the natural course of CPFE.

All clinical diagnostic and imaging evaluations in this study were performed by a multidisciplinary team consisting of two respiratory specialists and one radiologist. The final diagnosis was determined by comprehensive evaluation of clinical manifestations and imaging results.

### 2.5. Data Collection

Data from each patient were retrospectively extracted from the electronic medical records, including baseline clinical characteristics, the initial laboratory examinations when diagnosed firstly, pulmonary lung function, pathological type, location, and staging of the cancer. Moreover, the epidermal growth factor receptor (EGFR) mutations of adenocarcinoma were also included. Baseline information included age, gender, body mass index (BMI), smoking history, and underlying diseases. Secondly, laboratory examinations included white blood cell count (WBC), absolute neutrophil count (ANC), absolute lymphocyte count (ALC), platelet count (PLT), CRP, interleukin-6 (IL-6), fibrinogen, and complement C3 (C3). Smoking levels were expressed in pack-years, calculated by multiplying the number of packs of cigarettes consumed per day by the number of years smoked. The severity of pulmonary fibrosis was evaluated by using the composite physiological index (CPI). The formula for calculating CPI was 91 − (0.65 × DLCO%) − (0.53 × FVC%) + (0.34 × FEV1%). The neutrophil-to-lymphocyte ratio (NLR) was calculated by dividing the neutrophil count by the lymphocyte count. The platelet-to-lymphocyte ratio (PLR) was calculated by dividing the platelet count by the lymphocyte count.

All patients were followed up by telephone and outpatient or inpatient visits in November 2022. The median follow-up duration was 49.5 months (range: 3.4–84.2 months). Overall survival (OS) was calculated using the first day of treatment initiation as the event starting point, the last follow-up date as the event endpoint for surviving patients and patients lost to follow-up, and the date of death as the event endpoint for patients who died of any cause. The occurrence of AE, death, and the main cause of death were recorded during the follow-up period.

## 3. Statistical Analysis

Quantitative variables that approximately obeyed normal distribution were expressed as means and standard deviations, and the data were compared by one-way ANOVA test and *t*-test; those with non-normal distribution were expressed as median and interquartile range (M (IQR)), compared by Kruskal–Wallis H test and Mann–Whitney U test. Qualitative variables were expressed as frequencies and percentages, compared by chi-square test or Fisher’s exact test. Univariate survival analyses were performed using the Kaplan–Meier method and log-rank test, and survival curves were plotted. Cox proportional hazards regression was used to assess the effects of laboratory parameters and clinical characteristics on OS. Binary logistic regression analysis was performed to identify independent factors associated with death due to AE. Variables with *p* < 0.1 in univariate analysis were included in multivariate analysis. Two-tailed *p* < 0.05 was considered statistically significant. Software of SPSS 26.0 (SPSS Inc., Chicago, IL, USA) and the R program (version 4.2.0) was used for statistical analysis in this study.

## 4. Results

### 4.1. Clinical Characteristics in Patients with Lung Cancer

Clinical characteristics and tumor features of LC patients were shown in Table 1. Both IPF and emphysema were more common in elderly men and heavy smokers. The levels of WBC, CRP, IL-6, and fibrinogen were significantly higher in LC patients with IPF than in those with normal lungs. The pulmonary function tests in patients with emphysema solely suggested obstructive ventilation dysfunction characterized by a significant decrease in FEV1/FVC and relatively normal diffusion function, whereas patients with IPF mainly showed obvious diffusion dysfunction, accompanied by a significant increase in CPI and a noticeable reduction in the percent predicted diffusing capacity of the lung for carbon monoxide (DLCO). Unlike COPD with LC, IPF-associated cancer was more likely to occur in the lower lobe of the lung (56.0% vs. 33.3%, *p* = 0.001). The most common histological types in all three groups were adenocarcinoma and squamous cell carcinoma, and the majority of patients had advanced tumors at the time of initial diagnosis, especially patients with IPF. Notably, the proportion of adenocarcinoma was statistically lower in patients with IPF compared to patients with normal lungs (41.7% vs. 58.4%, *p* = 0.041), while squamous cell carcinoma appeared to be more prevalent, although there was no significant statistical difference.

### 4.2. Comparison Between CPFE and Isolated IPF in LC Patients

In the present research, 103 patients (68.2%) with IPF and LC were categorized as CPFE. Compared with the isolated IPF group, patients in the CPFE group were more prevalent as male smokers, especially heavy ones. The levels of serum CRP (30.2 vs. 12.9 mg/L, *p* = 0.031) and fibrinogen (5.08 vs. 4.38 g/L, *p* = 0.009) in patients with CPFE were significantly higher than those with isolated IPF, with no statistical difference in terms of NLR, PLR, and IL-6. Pulmonary function tests showed that the percent predicted of vital capacity or FEV1 was almost normal in the CPFE group, and FEV1/FVC was significantly lower than that in IPF patients without emphysema (76.3% vs. 80.7%, *p* = 0.004). No statistically significant differences were observed in the age, CPI, DLCO, localization, histological type, and pathological stage between patients in the CPFE and IPF-only groups (Table 2).

The results showed that there was no statistically significant difference in the overall incidence of AEs between the two groups (65.0% vs. 54.2%, *p* = 0.136). The incidence of treatment-related AE in the CPFE group was 49.5% (95%CI: 41.2–57.8%), which was significantly higher than 29.2% (95%CI: 21.5–37.8%) in the IPF group (*p* = 0.038), whereas no significant difference was observed about AE related to natural progression of CPFE between the two groups (15.5% vs. 25.0%, *p* = 0.589). In the CPFE-LC group, mortality analysis demonstrated the following distribution: advanced lung cancer constituted the predominant cause of death (48.0%), followed by adverse events (28.0%), severe pneumonia (20.0%), asphyxia (2.0%), and myocardial infarction (2.0%). In the IPF-LC cohort, mortality analysis revealed lung cancer as the leading cause of death (58.8%), followed by severe pneumonia with septic shock (23.5%), adverse events (11.8%), and acute pulmonary embolism (5.9%). More importantly, the mortality associated with AE in CPFE-LC patients was significantly higher than in the isolated IPF-LC group (28.0% vs. 11.8%, *p* = 0.045) (Table 3).

### 4.3. Comparison of EGFR Mutations Among Different Groups

A total of 132 adenocarcinoma patients underwent EGFR gene testing, including 49, 30, and 53 patients combined with IPF, emphysema, and normal lung in chest HRCT, respectively. Results showed that the EGFR mutation rate was obviously lower in adenocarcinoma patients with IPF compared to those with normal lungs (16.3% vs. 50.9%, *p* = 0.000). Considering the effect of smoking, the association between imaging features and EGFR mutations in 44 never-smokers and 88 ex- or current smokers was determined. The proportion of EGFR mutations was significantly lower in the IPF-LC group than the Norm-LC group (8.8% and 40.7%, *p* = 0.003) in ex- or current smokers. Among never smokers, there was no statistically significant difference in the incidence of EGFR mutations in the three groups (Table 4).

Furthermore, no statistical difference in the prevalence of EGFR mutations was found between the CPFE-LC and isolated IPF-LC groups (6/36, 16.7% vs. 2/13, 15.4%, *p* = 0.915).

### 4.4. Overall Survival Analysis in All Patients and in IPF-LC Patients with AE

**Figure 1:**
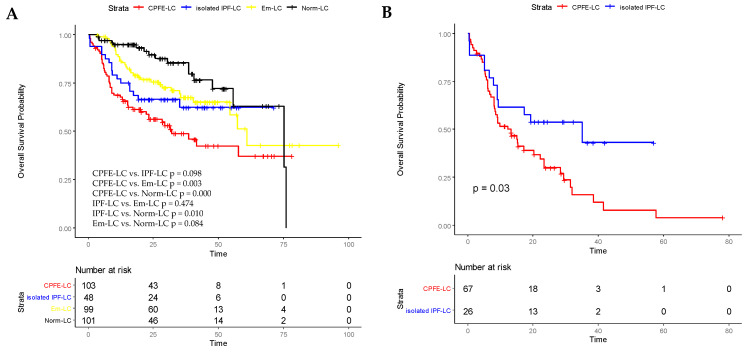
The Kaplan–Meier analyses of OS of the four groups were shown in (**A**) (*p*-values for OS of the CPFE-LC group vs. Em-LC and Norm-LC groups: 0.003 and 0.000, respectively; *p*-values for OS of the CPFE-LC group vs. IPF-LC groups: 0.098; *p*-values of the Em-LC group vs. Norm-LC group: 0.084). The Kaplan–Meier survival curve of patients with IPF and lung cancer who developed AE was shown in (**B**). OS, overall survival; CPFE-LC: lung cancer patients with idiopathic pulmonary fibrosis and emphysema; isolated IPF-LC: lung cancer patients with idiopathic pulmonary fibrosis and without emphysema; Em-LC: patients with emphysema and lung cancer; Norm-LC: lung cancer patients with normal lungs.

Kaplan–Meier survival curves showed that overall survival was significantly shorter in the CPFE-LC group compared with the Em-LC group (32.9 vs. 61.0 months, *p* = 0.003, log-rank test) and Norm-LC group (32.9 vs. 75.1 months, *p* = 0.000, log-rank test). There was no significant difference in the survival between the CPFE-LC and isolated IPF-LC groups (32.9 vs. 42.8 months, *p* = 0.098, log-rank test). The 1-, 3-, and 5-year survival rates in the CPFE-LC group were 65.0%, 16.5%, and 5.8%, respectively. The 3-year survival rates were 16.5%, 29.2%, 36.4%, and 30.7% in the CPFE, solely IPF, emphysema, and normal LC patients, respectively. In addition, the Kaplan–Meier survival curve showed the survival was significantly shorter in the IPF-alone group than in those with normal lungs (*p* = 0.010, log-rank test). No statistical difference was observed between the isolated IPF and solely emphysema groups (*p* = 0.474, log-rank test) (Figure 1A).

Meanwhile, in this study, a subgroup analysis was performed for IPF and LC patients who developed AE. The median survival was 19.4 months (95%CI: 14.0–24.7 months) in the CPFE-LC group compared with 31.7 months (95%CI: 22.1–41.4 months) in the isolated IPF-LC group. The Kaplan–Meier survival curve indicated that among patients who developed AE, overall survival was significantly shorter in LC patients with CPFE than in those with solely IPF (*p* = 0.030, log-rank test) (Figure 1B).

### 4.5. Prognostic Variables for Overall Survival in Patients with Lung Cancer

Among patients with LC, univariate regression analysis revealed that age ≥ 65 years, male, ex- or current smokers, pack-years ≥ 20, high level of fibrinogen, CPI ≥ 40, tumor EGFR wild-type, advanced lung cancer, and coexistence of COPD, IPF, or CPFE were related to poor survival. After multivariate regression analysis, it was demonstrated that CPI ≥ 40 (HR: 2.087, 95% CI: 1.715–6.089, *p* = 0.012), coexistence of COPD (HR: 2.281, 95% CI: 1.139–4.569, *p* = 0.040), isolated IPF (HR: 5.703, 95% CI: 2.516–12.925, *p* = 0.000), or CPFE (HR: 6.275, 95%CI: 3.379–11.652, *p* = 0.000) were independent risk factors of poor prognosis after adjusting for age, sex, smoking history, EGFR mutation, and tumor stage (Table 5).

### 4.6. CPFE as a Predictor Associated with AE-Related Mortality in Patients with IPF and LC

The factors correlated with death due to AE were investigated in LC patients with IPF. Binary logistic regression analysis displayed that CPFE (OR: 3.494, 95% CI: 2.014–6.063, *p* = 0.001) was remarkably associated with enhanced AE-related mortality in patients with LC and IPF (Table 6).

## 5. Discussion

The present study had several important findings. First, the prognosis of CPFE and IPF combined with LC was significantly worse, which may be related to more explosive systemic inflammation, lower EGFR mutation rate, fewer treatment options, and increased risk of treatment-induced AE. More importantly, although compared to LC patients with solely IPF, the presence of emphysema did not reduce overall survival, CPFE increased the risk of treatment-related AE and was independently associated with AE-related mortality. At last, in patients with LC, AEs with CPFE had a worse prognosis than those with IPF.

Multiple previous studies have demonstrated a high prevalence of CPFE among patients with IPF [7,8,25]. For example, USUI et al. found that of the 116 LC patients with IPF included, up to 101 patients (87%) were categorized in the CPFE group [12]. In the present research, 103 patients (68.2%) of IPF patients combined with LC were identified as CPFE. The higher incidence of CPFE in IPF patients with LC may be correlated with a significantly elevated risk of developing LC in CPFE patients. Consistent with the results of previous descriptive studies [14,16,26], in our research, CPFE combined with LC was common in older male smokers. Lung cancer in CPFE was prone to occur in the lower lobes (59.2%) and adjacent fibrotic areas (57.3%), similar to IPF alone combined with lung cancer. Although adenocarcinoma was still the most common, the proportion of squamous carcinoma in CPFE combined with LC increased compared to patients with normal lungs. Meanwhile, compared to patients with emphysema and normal lungs, patients with CPFE had higher levels of serum inflammation, characterized by significantly elevated levels of WBC, CRP, IL-6, and fibrinogen. Under the effect of exposure factors such as smoking or dust, airway epithelial cells release inflammatory mediators and further synthesize IL-6 and CRP, creating an exaggerated inflammatory waterfall, leading to injury of alveoli and promoting the development, progression, and poor prognosis of cancer [2].

In recent years, it was reported that EGFR mutations were associated with longer survival in advanced adenocarcinoma [27]. Molecular targeted therapy represented by epidermal growth factor receptor tyrosine kinase inhibitor (EGFR-TKI) can significantly prolong the overall survival (OS) of patients and has been approved for the first-line treatment of advanced NSCLC patients with positive EGFR gene mutation [28,29,30,31]. Notably, observational studies suggest that COPD is independently associated with reduced rates of EGFR mutations and ALK rearrangements, with mutation frequency inversely correlated to airway obstruction severity. Consistent with this, survival analysis revealed that NSCLC patients with COPD receiving EGFR-TKI therapy had shorter progression-free survival (PFS) and poorer survival outcomes compared to non-COPD patients [32]. We hypothesize that the COPD-associated chronic inflammatory microenvironment (e.g., elevated IL-6 or TNF-α levels) may impair the efficacy of EGFR-TKI therapy through activation of the JAK/STAT signaling pathway, a hypothesis requiring experimental validation. In addition, Daichi Fujimoto’s study has similarly described a significantly reduced frequency of EGFR mutations in patients with ILD and lung adenocarcinoma [33]. They formulated that carrying EGFR mutations was obviously associated with the absence of ILD in patients with lung adenocarcinoma (OR: 17.41, 95% CI: 3.54–315.34, *p* < 0.001), independent of gender and smoking status. Although limited by small sample size, this preliminary finding raises the hypothesis that the coexistence of fibrotic and emphysematous microenvironments may synergistically inhibit canonical driver mutations, favoring alternative oncogenic pathways. Future large-scale sequencing studies are required to validate this hypothesis and delineate CPFE-specific genomic signatures [34].

In this study, the EGFR mutation rate was significantly lower in patients with IPF combined with adenocarcinoma. Although the proportion of IPF patients in men and smokers was higher than that in non-IPF patients, only 8.8% of IPF patients carried EGFR mutations in male smokers, which was significantly different from patients without IPF. Although no significant statistical difference was found due to small samples, the lower EGFR mutation rate of IPF patients was also observed in non-smokers, suggesting that the presence of IPF itself was negatively correlated with tumor EGFR mutation, which was similar to previous reports. This may be partly responsible for fewer treatment options and poor prognosis of CPFE-LC patients. The specific mechanism of this negative correlation has not been clear, which may be related to the carcinogenic mechanism of IPF patients being different from EGFR mutation patients [2,29,35,36]. These findings suggest that pulmonary fibrosis and emphysema may have certain effects on tumor-related gene expression and pathways, gene variation, and so on. In the future, the correlation between CT manifestations and gene expression and prognosis should be further explored for better guiding the clinic.

Compared to patients without CPFE, CPFE is associated with poor survival [37]. Hajime OTSUKA et al. reported the poor survival of CPFE patients after surgical resection, with 3-year and 5-year survival rates of 38% and 22%, respectively [16]. Our survival analysis indicated that 1-, 3-, and 5-year survival rates in the CPFE-LC group were 65.0%, 16.5%, and 5.8%, respectively, which may reflect differences in sample size or tumor staging of the involved patients. These poor prognostic outcomes can be explained, at least in part, by the increased incidence of cancer and treatment-related mortality in CPFE, which often limits standard treatment. A relatively large number of patients with CPFE are unsuitable for all forms of therapy, and those who are treated typically have a higher incidence of complications such as acute lung injury, acute exacerbations, and tumor recurrence, which may result from the severe damage to lung tissue caused by the combination of emphysema and fibrosis [7].

There were no significant differences in tumor features, EGFR mutations, and overall survival between CPFE patients and isolated IPF patients with LC in this study, which was in accordance with Yuji Minegishi et al.’s study [14]. They found that CPFE was not an independent prognostic factor in LC patients with IIPs. Likewise, OTSUKA’s research reported that the 5-year survival rate for surgically resected lung cancer patients with CPFE was very poor and not statistically different from LC patients with IPF alone. Moon and his co-workers formulated no association between CPFE and higher mortality in univariate (HR: 1.00; 95% CI: 0.75–1.32, *p* = 0.972) or multivariate analyses (HR: 0.89; 95% CI: 0.66–1.21, *p* = 0.466) in patients with IPF and non-small cell lung cancer (NSCLC) [15]. Previous studies have shown that the presence or extent of emphysema does not affect the survival of patients with IPF after correction for baseline severity of pulmonary fibrosis and emphysema. These results suggested that pulmonary fibrosis may play a more important role in the pathophysiological mechanism of pathogenesis in CPFE with LC and that CPFE and isolated IPF may act through similar tumorigenesis and progression mechanisms.

The risk of AE associated with IPF treatment is particularly noteworthy in CPFE patients. Our study showed that the presence of emphysema increases the risk of treatment-induced AE, such as chemotherapy and surgery, and AE-related lethality. More importantly, in patients with LC, the occurrence of AE in CPFE had a worse prognosis than the occurrence of AE in IPF. Otsuka et al. reported that postoperative-related AE occurred in 13.0% of patients with surgically resected lung cancer combined with CPFE, and all of them died of AE during follow-up [16]. In Moon’s study, AE was more common in NSCLC patients with CPFE within 1 month after treatment (chemotherapy, surgery, or radiotherapy) than in patients with IPF alone. They found that CPFE was significantly associated with AE in patients with NSCLC [15]. Jee Youn Oh et al. found that up to 22.0% of patients with LC combined with CPFE died from AE [21]. In the present study, 48.5% of CPFE patients died during the follow-up period, 28.0% of which were due to AE, significantly higher than in patients with IPF and LC. AEs of IPF are usually significantly associated with poor prognosis. Therefore, AE prevention is critical, especially in the CPFE population. A clinical trial of pirfenidone in AEs of IPF showed significantly better 3-month survival in patients treated with pirfenidone than in controls [38]. More importantly, Cottin et al., in a nintedanib INPULSIS trial, found no difference in the treatment effect for the presence of mild to moderate emphysema in patients with IPF [39]. The judicious use of anti-fibrotic drugs such as nintedanib and pirfenidone, careful selection of anti-tumor treatment such as preservation of lung resection and targeted drug therapy, and so on have the potential to provide a good prognosis and prevention of AEs in patients with IPF and LC [2,40,41]. Physicians should carefully monitor patient status. Clinical information and chest HRCT should be used to adequately identify those at high risk of developing AEs and to diagnose AEs in a timely manner so as to provide early intervention for AEs. Although the present study did not find a significant effect of smoking status or fibrosis severity on survival after AE in patients with CPFE (possibly due to sample size limitations), smoking, as a common risk factor for IPF and lung cancer, may exacerbate disease progression by enhancing the inflammatory microenvironment. Future studies need to expand the sample size to further investigate the association of these factors with prognosis in patients with CPFE combined with lung cancer.

Emphysema and pulmonary fibrosis have opposite physiological effects. Emphysema is characterized by reduced elastic recoil, increased compliance, and enhanced lung volume, whereas fibrosis leads to reduced elastic recoil, compliance, and lung volume. The coexistence of fibrosis and emphysema results in preserved air flow and lung volume, but with significant limitations in gas exchange. In order to avoid the interference of the thickness and turbidity of emphysema and its influence on lung function, Wells et al. proposed to evaluate the severity of fibrosis by using the composite physiological index (CPI) in patients with pulmonary fibrosis and emphysema, which can provide more powerful prognostic information and more accurate overall assessment than individual pulmonary function parameters [42,43]. Our study revealed that CPI ≥ 40 were significant predictors of mortality after adjusting for age, sex, smoking history, and LC stage, which is consistent with the findings of Li et al. They identified CPI as an independent risk factor for mortality in patients with IPF and emphysema (IPFE) after adjusting for the percent of pulmonary fibrosis in HRCT and DLCO (%pred) [44]. Fumika Ueno et al. reviewed CPFE patients with LC who underwent surgery and found that high preoperative CPI was independently associated with a high risk of mortality [45]. The statistical difference was observed in the overall survival difference between the CPI > 41 group and the CPI < 41 group. It has been demonstrated that CPI can provide more powerful prognostic information and more accurate overall assessment than individual pulmonary function parameters [37,38].

This study has several limitations that should be acknowledged: (1) Retrospective single-center design: The reliance on a single-center, small-sample retrospective analysis may introduce selection bias and reduce statistical power. (2) Subjective imaging assessment: Emphysema and fibrosis were evaluated using visual scoring rather than standardized quantitative software, which may increase interobserver variability and measurement bias. (3) Lack of quantified lesion stratification: The severity of emphysema and fibrosis was not quantitatively stratified, restricting further exploration of dose-response relationships between imaging phenotypes and clinical outcomes. Despite these limitations, to our knowledge, this was the first study to focus on the effect of CPFE on the AE-related lethality and poor prognosis in LC patients who developed AE. Future multicenter prospective studies or international collaborative cohorts should be conducted to validate these findings, incorporating standardized quantitative imaging methodologies (e.g., AI-based analytical approaches) to enhance both the generalizability and precision of the conclusions. The AE early warning model and anti-inflammatory intervention strategies for patients with CPFE-LC could also be developed to improve prognosis. In-depth exploration of the molecular mechanisms (e.g., inflammation-fibrosis interaction pathways) driving LC progression and AE in the CPFE microenvironment is also essential.

## 6. Conclusions

The prognosis of CPFE and IPF combined with LC was significantly worse, which may be related to a more explosive systemic inflammatory response, a lower EGFR mutation rate, less choice of treatment, and an increased risk of AE. Compared to LC patients with solely IPF, the presence of emphysema had no significant impact on survival time; however, CPFE increased the risk of treatment-related AE and was associated with AE-related mortality. More importantly, in patients with LC, CPFE with AEs had a worse prognosis than IPF with AEs. Consequently, patients with CPFE-LC may require intensified surveillance protocols and personalized therapeutic strategies to reduce the risk of treatment-related acute exacerbations.

## Figures and Tables

**Table 1:** Clinical characteristics and tumor features of lung cancer patients in different groups.

	IPF-LC Group *n* = 151	Em-LC Group *n* = 99	Norm-LC Group *n* = 101	IPF-LC vs. Em-LC *p* Value	IPF-LC vs. Norm-LC *p* Value
Patient characteristics, number (%), or median (Q1–Q3)					
Age, years	68 (64–73)	66 (58–73)	60 (53–67)	0.167	0.000
Sex (men)	145/151 (96.0%)	92/99 (92.9%)	75/101 (74.3%)	0.582	0.000
Ex- or current smokers	128/151 (84.8%)	89/99 (89.9%)	59/101 (58.4%)	0.861	0.000
Pack-years	37.5 (19.5–50.0)	40.0 (20.0–50.0)	10.3 (5.0–30.0)	0.742	0.000
BMI, kg/m^2^	22.9 (20.6–24.9)	22.3 (20.4–24.2)	22.2 (20.4–24.1)	0.563	0.219
Dust exposure	4/151 (2.6%)	1/99 (1.0%)	0/101 (0.0%)	0.427	0.233
Previous pulmonary tuberculosis	12/151 (7.9%)	2/99 (2.0%)	3/101 (3.0%)	0.096	0.175
Laboratory examinations and pulmonary function parameters, median (Q1–Q3)					
WBC, ×10^9^/L	7.99 (6.42–10.38)	7.15 (5.49–9.51)	6.71 (5.84–8.89)	0.098	0.000
ANC, ×10^9^/L	5.63 (4.12–8.14)	4.93 (3.34–6.98)	4.67 (3.74–6.68)	0.284	0.100
ALC, ×10^9^/L	1.44 (1.08–1.90)	1.27 (0.96–1.97)	1.39 (1.04–1.79)	0.472	0.641
NLR	3.74 (2.49–6.47)	3.31 (2.12–5.57)	3.66 (2.46–5.14)	0.157	0.540
PLR	133.2 (92.6–200.7)	137.0 (83.3–213.7)	139.8 (94.7–177.4)	0.732	0.986
CRP, mg/L	52.7 (8.6–102.7)	9.37 (3.95–37.40)	18.9 (8.05–66.85)	0.012	0.024
IL-6, μg/L	32.82 (11.2–97.55)	10.72 (5.65–35.15)	13.69 (5.27–29.8)	0.020	0.039
Fibrinogen, g/L	4.94 (3.66–5.67)	4.56 (3.15–5.18)	4.13 (3.01–5.07)	0.134	0.019
C3, g/L	0.97 (0.85–1.09)	0.90 (0.80–0.98)	1.07 (0.94–1.21)	0.561	0.243
CPI	44.94 (30.88–46.97)	15.57 (10.52–19.13)	10.56 (7.38–15.61)	0.000	0.000
FVC, %pred	83.4 (67.5–97.7)	101.6 (80.1–120.9)	97.5 (89.4–104.2)	0.045	0.065
FEV1, %pred	90.5 (79.1–102.1)	68.6 (43.9–86.4)	99.5 (86.1–109.3)	0.000	0.490
FEV1/FVC, %	76.9 (71.4–82.6)	60.1 (48.3–68.4)	83.4 (75.6–87.7)	0.041	0.238
DLCO, %pred	56.8 (44.1–67.3)	77.5 (58.4–89.6)	102.0 (84.6–116.4)	0.000	0.000
Tumor characteristics, number (%)					
Localization				0.001	0.126
Upper lobe	22/50 (44.0%)	72/108 (66.6%)	53/102 (52.0%)		
Lower lobe	28/50 (56.0%)	36/108 (33.3%)	49/102 (48.0%)		
Pathological type				0.504	0.063
Adenocarcinoma	63/151 (41.7%)	40/99 (40.4%)	59/101 (58.4%)		0.041
Squamous carcinoma	56/151 (37.1%)	39/99 (39.4%)	23/101 (22.8%)		0.079
Others	32/151 (21.2%)	20/99 (20.2%)	19/101 (18.8%)		0.586
Staging				0.139	0.049
I–II	27/151 (17.9%)	25/99 (25.3%)	38/101 (37.6%)		
III–IV	124/151 (82.1%)	74/99 (74.7%)	63/101 (62.4%)		

IPF-LC: patients with idiopathic pulmonary fibrosis and lung cancer; Em-LC: patients with emphysema and lung cancer; Norm-LC: lung cancer patients with normal lungs; BMI: body mass index; WBC: white blood cell count; ANC: absolute neutrophil count; ALC: absolute lymphocyte count; NLR: neutrophil to lymphocyte ratio; PLR: platelet to lymphocyte ratio; CRP: C-reactive protein; IL-6: interleukin-6; C3: complement 3; CPI: compound physiological index; FVC: forced vital capacity; FEV1: forced expiratory volume in the first second; DLCO: diffusing capacity for carbon monoxide; Q: quartile.

**Table 2:** Comparison of the demographic and clinical characteristics of CPFE and isolated IPF groups in LC patients.

	CPFE-LC Group N = 103	Isolated IPF-LC Group N = 48	*p* Value
Patient characteristics, number (%), or median (Q1–Q3)			
Age, years	68 (63–73)	69 (64–74)	0.261
Sex (men)	103/103 (100.0%)	42/48 (87.5%)	0.001
Ex- or current smokers	93/103 (90.3%)	37/48 (77.1%)	0.043
Pack-years	40 (21.25–50)	27.5 (4.2–50)	0.026
Laboratory examinations and pulmonary function parameters, median (Q1–Q3)			
WBC, ×10^9^/L	7.77 (6.43–10.28)	8.85 (6.41–10.85)	0.391
ANC, ×10^9^/L	5.49 (4.08–7.67)	6.23 (4.28–8.32)	0.230
ALC, ×10^9^/L	1.45 (1.05–1.91)	1.42 (1.08–1.90)	0.691
NLR	3.62 (2.41–6.37)	3.99 (2.51–7.03)	0.358
PLR	129.6 (89.5–206.9)	151.6 (96.6–192.3)	0.324
CRP, mg/L	30.2 (9.36–103.50)	12.9 (5.31–78.3)	0.031
IL-6, μg/L	24.95 (10.71–91.25)	32.13 (9.88–59.77)	0.104
Fibrinogen, g/L	5.08 (4.02–5.83)	4.38 (3.53–5.17)	0.009
C3, g/L	1.02 (0.85–1.10)	0.93 (0.86–1.08)	0.847
CPI	46.68 (26.88–56.64)	45.24 (21.35–56.67)	0.112
VC, %pred	90.0 (79.2–97.7)	85.2 (67.9–102.3)	0.201
FVC, %pred	83.4 (71.5–100.6)	92.6 (60.7–95.6)	0.943
FEV1, %pred	89.5 (78.6–103.4)	92.6 (80.2–100.5)	0.716
FEV1/FVC, %	76.3 (68.8–80.7)	80.7 (76.6–85.43)	0.004
DLCO, %pred	57.4 (47.1–68.4)	50.6 (39.9–63.6)	0.085
Tumor characteristics, number (%)			
localization			0.315
Upper lobe	42/103 (40.8%)	22/48 (45.8%)	
Lower lobe	61/103 (59.2%)	26/48 (54.2%)	
Cancer in fibrosis areas	59/103 (57.3%)	33/48 (68.8%)	0.214
Pathological type			0.451
Adenocarcinoma	43/103 (41.7%)	20/48 (41.7%)	
Squamous carcinoma	37/103 (35.9%)	19/48 (39.6%)	
Others	23/103 (22.4)	9/48 (18.8%)	
Staging			0.170
I–II	15/103 (14.6%)	12/48 (25.0%)	
III–IV	88/103 (85.4%)	36/48 (75.0%)	

CPFE-LC: lung cancer patients with idiopathic pulmonary fibrosis and emphysema; isolated IPF-LC: lung cancer patients with idiopathic pulmonary fibrosis and without emphysema; BMI: body mass index; WBC: white blood cell count; ANC: absolute neutrophil count; ALC: absolute lymphocyte count; NLR: neutrophil to lymphocyte ratio; PLR: platelet to lymphocyte ratio; CRP: C-reactive protein; IL-6: interleukin-6; C3: complement 3; CPI: compound physiological index; VC: vital capacity; FVC: forced vital capacity; FEV1: forced expiratory volume in the first second; DLCO: diffusing capacity for carbon monoxide; AE: acute exacerbation; Q: quartile.

**Table 3:** Comparison of the incidence of acute exacerbations between the CPFE group and the isolated IPF group in lung cancer patients.

	CPFE-LC Group N = 103	Isolated IPF-LC Group N = 48	*p* Value
AE	67/103 (65.0%)	26/48 (54.2%)	0.136
Treatment-induced AE	51/103 (49.5%)	14/48 (29.2%)	0.038
Natural course associated AE	16/103 (15.5%)	12/48 (25.0%)	0.589
Mortality	50/103 (48.5%)	17/48 (35.4%)	0.095
Main cause of death			
Lung cancer progression	24/50 (48.0%)	10/17 (58.8%)	0.736
Acute exacerbation	14/50 (28.0%)	2/17 (11.8%)	0.045
Severe pneumonia	10/50 (20.0%)	4/17 (23.5%)	0.684
Asphyxia	1/50 (2.0%)	0/17 (0.0%)	0.848
Acute pulmonary embolism	0/50 (0.0%)	1/17 (5.9%)	0.073
Acute myocardial infarction	1/50 (2.0%)	0/17 (0.0%)	0.848

**Table 4:** Comparison of EGFR mutation between different groups of adenocarcinoma patients.

	IPF-LC Group	Em-LC Group	Norm-LC Group	IPF-LC vs. Em-LC *p* Value	IPF-LC vs. Norm-LC *p* Value
In adenocarcinoma patients	8/49 (16.3%)	5/30 (16.7%)	27/53 (50.9%)	0.899	0.000
In ex- or current smokers	3/34 (8.8%)	3/27 (11.1%)	11/27 (40.7%)	0.316	0.003
In never smokers	5/15 (33.3%)	2/3 (66.7%)	16/26 (61.5%)	0.079	0.092

IPF-LC: patients with idiopathic pulmonary fibrosis; Em-LC: patients with emphysema and lung cancer; Norm-LC: lung cancer patients with normal lungs; EGFR: epidermal growth factor receptor mutation.

**Table 5:** Cox regression prognostic variables for overall survival in patients with lung cancer.

Variable	Univariate Analyses		Multivariate Analyses	
	HR (95% CI)	*p* Value	HR (95% CI)	*p* Value
Sex: women	Re			
men	2.747 (1.276–5.915)	0.010		
Age <65 years	Re			
≥65 years	1.023 (1.002–1.044)	0.030		
Smoking history: Never	Re			
Ex- or current smokers	2.481 (1.416–4.347)	0.002		
Pack-years < 20	Re			
≥20	2.416 (1.191–4.903)	0.015		
WBC, ×10^9^/L	0.992 (0.987–1.007)	0.181		
CRP, mg/L	1.004 (0.999–1.009)	0.057		
Fibrinogen, g/L	1.187 (1.047–1.345)	0.025		
CPI < 40	Re			
CPI ≥ 40	1.978 (1.049–6.718)	0.034	2.087 (1.715–6.089)	0.012
Staging I–II	Re			
III–IV	3.121 (1.704–5.715)	0.039		
EGFR mutation	Re			
EGFR wild-type	2.247 (1.175–4.297)	0.014		
Normal Lung	Re			
COPD	2.196 (1.120–4.304)	0.038	2.281 (1.139–4.569)	0.040
Isolated IPF	4.270 (2.455–7.426)	0.022	5.703 (2.516–12.925)	0.000
CPFE	4.320 (2.007–9.299)	0.000	6.275 (3.379–11.652)	0.000

HR: hazard ratio; CI: confidence interval; WBC: white blood cell count; CRP: C-reactive protein; CPI: compound physiological index; EGFR: epidermal growth factor receptor; COPD: chronic obstructive pulmonary disease; IPF: idiopathic pulmonary fibrosis; CPFE: combined pulmonary fibrosis and emphysema; Re: reference.

**Table 6:** Factors associated with death due to AE in patients with IPF and LC.

Variable	Univariate Analyses		Multivariate Analyses	
	OR (95% CI)	*p* Value	OR (95% CI)	*p* Value
Sex: women	Re			
men	1.888 (0.182–2.164)	0.151		
Smoking history: never	Re			
Ex- or current smokers	1.164 (0.991–1.827)	0.854		
Pack-years < 20	Re			
Pack-years ≥ 20	2.906 (1.562–5.406)	0.573		
Fibrinogen, g/L	2.481 (1.416–3.347)	0.044		
CRP, mg/L	1.004 (0.999–1.009)	0.102		
Non-CPFE	Re			
CPFE	2.996 (1.120–4.304)	0.030	3.494 (2.014–6.063)	0.001

OR: odds ratio; CI: confidence interval; CRP: C-reactive protein; CPFE: combined pulmonary fibrosis and emphysema; IPF: idiopathic pulmonary fibrosis; Re: reference; AE: acute exacerbation.

## Data Availability

The datasets used and/or analyzed during the current study are available from the corresponding author on reasonable request.
